# Role of Ferrous Sulfate (FeSO_4_) in Resistance to Cadmium Stress in Two Rice (*Oryza sativa* L.) Genotypes

**DOI:** 10.3390/biom10121693

**Published:** 2020-12-18

**Authors:** Javaria Afzal, Muhammad Hamzah Saleem, Fatima Batool, Ali Mohamed Elyamine, Muhammad Shoaib Rana, Asma Shaheen, Mohamed A. El-Esawi, Muhammad Tariq Javed, Qasim Ali, Muhammad Arslan Ashraf, Ghulam Sabir Hussain, Chengxiao Hu

**Affiliations:** 1 Key Laboratory of Arable Land Conservation (Middle and Lower Reaches of Yangtze River), Ministry of Agriculture and Rural Affairs, Huazhong Agricultural University, Wuhan 430070, China; juvaria_afzal@outlook.com (J.A.); muhammadshoaib1555@gmail.com (M.S.R.); 2 Department of Soil Science, Sindh Agriculture University, Tandojam 70060, Pakistan; 3 College of Plant Science and Technology, Huazhong Agricultural University, Wuhan 430070, China; saleemhamza312@webmail.hzau.edu.cn; 4 Department of Botany, Division of Science and Technology, University of Education Lahore, Punjab 54770, Pakistan; fatima.batool@ue.edu.pk; 5 College of Science, Shantou University, Shantou 515063, China; elyoh@hotmail.fr; 6 Department of Earth Sciences, University of Sargodha, Sargodha 40100, Pakistan; neil_kanth@yahoo.com; 7 Botany Department, Faculty of Science, Tanta University, Tanta 31527, Egypt; mohamed.elesawi@science.tanta.edu.eg; 8 Department of Botany, Government College University, Faisalabad 38000, Pakistan; mtariqjaved@gcuf.edu.pk (M.T.J.); drqasimali@gcuf.edu.pk (Q.A.); marslanashraf@gcuf.edu.pk (M.A.A.); 9 Department of Agronomy, Bahauddin Zakariya University, Multan 60800, Pakistan; hussainsabirsial90@yahoo.com; 10 Department of Technical Services, Fatima Agri Sales and Services, Multan 60800, Pakistan

**Keywords:** antioxidants, defense mechanism, heavy metals, iron, organic acids exudation

## Abstract

The impact of heavy metal, i.e., cadmium (Cd), on the growth, photosynthetic pigments, gas exchange characteristics, oxidative stress biomarkers, and antioxidants machinery (enzymatic and non-enzymatic antioxidants), ions uptake, organic acids exudation, and ultra-structure of membranous bounded organelles of two rice (*Oryza sativa* L.) genotypes (Shan 63 and Lu 9803) were investigated with and without the exogenous application of ferrous sulfate (FeSO_4_). Two *O. sativa* genotypes were grown under different levels of CdCl_2_ [0 (no Cd), 50 and 100 µM] and then treated with exogenously supplemented ferrous sulfate (FeSO_4_) [0 (no Fe), 50 and 100 µM] for 21 days. The results revealed that Cd stress significantly (*p* < 0.05) affected plant growth and biomass, photosynthetic pigments, gas exchange characteristics, affected antioxidant machinery, sugar contents, and ions uptake/accumulation, and destroy the ultra-structure of many membranous bounded organelles. The findings also showed that Cd toxicity induces oxidative stress biomarkers, i.e., malondialdehyde (MDA) contents, hydrogen peroxide (H_2_O_2_) initiation, and electrolyte leakage (%), which was also manifested by increasing the enzymatic antioxidants, i.e., superoxidase dismutase (SOD), peroxidase (POD), catalase (CAT) and ascorbate peroxidase (APX) and non-enzymatic antioxidant compounds (phenolics, flavonoids, ascorbic acid, and anthocyanin) and organic acids exudation pattern in both *O. sativa* genotypes. At the same time, the results also elucidated that the *O. sativa* genotypes Lu 9803 are more tolerant to Cd stress than Shan 63. Although, results also illustrated that the exogenous application of ferrous sulfate (FeSO_4_) also decreased Cd toxicity in both *O. sativa* genotypes by increasing antioxidant capacity and thus improved the plant growth and biomass, photosynthetic pigments, gas exchange characteristics, and decrease oxidative stress in the roots and shoots of *O. sativa* genotypes. Here, we conclude that the exogenous supplementation of FeSO_4_ under short-term exposure of Cd stress significantly improved plant growth and biomass, photosynthetic pigments, gas exchange characteristics, regulate antioxidant defense system, and essential nutrients uptake and maintained the ultra-structure of membranous bounded organelles in *O. sativa* genotypes.

## 1. Introduction

Metal contamination issues are becoming increasingly common in China and elsewhere, with many documented cases of metal toxicity in mining industries, foundries, smelters, coal-burning power plants, and agriculture [1,2,3,4,5]. Heavy metal accumulation in soils is of great concern in agricultural production due to its adverse effects on food safety and marketability, crop growth due to phytotoxicity, and the environmental health of soil organisms [6,7,8,9,10]. In addition, heavy metal contamination of soil may pose risks and hazards to humans and the ecosystem through: Direct ingestion or contact with contaminated soil, the food chain (soil-plant-human or soil-plant-animal human), drinking of contaminated groundwater, reduction in food quality (safety and marketability) via phytotoxicity, reduction in land usability for agricultural production causing food insecurity, and land tenure problems [11,12]. Heavy metals include cadmium (Cd), lead (Pb), nickel (Ni), cobalt (Co), iron (Fe), zinc (Zn), chromium (Cr), iron (Fe), arsenic (As), silver (Ag) and the platinum group elements. Contamination of agricultural soils with Cd has become one of the most toxic and widespread environmental problems [13]. Cd is mainly entered into the ecosystem through human activities such as agricultural practices and mining activities [14,15]. The regulatory limit of Cd in agricultural soil is 100 mg kg^−1^ soil [10]. Photosynthesis, respiration, cell division, water relations, opening and closing of stomata, nitrogen metabolism, and mineral nutrition are the main metabolic processes within the plants, which are negatively affected by Cd stress [16,17]. Although Cd is toxic for plant growth, it is easily taken by the roots and then transported to the shoots where it can cause retorted growth, stunted root development, reduce branching, alteration in photosynthesis and respiration, diminished nutrient uptake, blocked electron transport chain as well as changed the membrane permeability [1,18,19,20,21,22]. The inhibition of root Fe(III) reductase induced by Cd led to Fe(II) deficiency, and it seriously affected photosynthesis [10,23]. Moreover, higher Cd retention in plant cells/tissues triggers the production of reactive oxygen species (ROS), hydroxyl groups (OH), and superoxide radicles (O^·−^), which either directly or indirectly affects the in planta metabolic pathways [14,24,25]. Over-production of ROS is toxic, and plants need to scavenge those immediately through an antioxidative defense system [14,26]. Previously, antioxidative enzymes played a significant role in the reduction of Cd phytotoxicity in *Glycine max* [21], *Solanum lycopersicum* [27], *Pfaffia glomerata* [28], *Oryza sativa* [29], *Boehmeria nivea* [20], and *Zea mays* [30] grown under excessive Cd concentrations.

Heavy metals are natural components of the terrestrial ecosystem. However, their presence in excess is harmful to humans and the environment. Therefore, remediation is necessary to alleviate the negative effects caused by the heavy metals incorporated into ecosystems [3,31,32]. Research has been continuing to develop effective methods of remediation to treat contaminated lands. Remediation could be done by immobilization, removal, sequestration, active mixing, and phytoextraction [33,34,35]. However, selection and applicability of the remediation methods depend on a number of factors, including cost, duration of effectiveness, commercial level availability, general acceptance, applicability to high metal contents, and applicability to mixed metal and organic wastes as well as toxicity, mobility, and volume reduction [36,37]. Heavy metal toxicity can be minimized by reducing their availability using organic and inorganic amendments [12,38]. Different types of iron (Fe) fertilizer can have distinct effects on Cd accumulation [39]. For example, Shao et al. [40] found that the application of chelated Fe (EDTANa_2_Fe) markedly decreased Cd concentration in shoots, roots, and grain, while the application of ionic Fe (FeSO_4_) significantly enhanced the Cd concentration in shoots and roots. Thus, the selection of appropriate Fe fertilizer and application methods is important for improving growth and reducing Cd accumulation in rice [41]. On the other hand, Fe is an essential micronutrient, and its deficiency can reduce the growth, yield, and nutritional quality of agricultural crops worldwide [38,42]. Many studies have used soil-applied Fe fertilizer to improve plant growth and Cd metabolism in crops [38,43], which may be affected by pH and Eh conditions, antagonism among nutrients, and other confounding chemistries occurring in soils. In addition, previous studies have demonstrated that the application of Fe fertilizer can be more effective for enhancing yield and photosynthesis in crops [23,44]. However, few studies have compared the efficiency of chelate vs. ion and ferrous vs. ferric Fe fertilizer on Cd mitigation in *Oryza sativa* [45,46]. 

Rice (*Oryza sativa* L.) is the seed of the grass species *Oryza glaberrima* (African rice) or *Oryza sativa* (Asian rice), and as a cereal grain, it is the most widely consumed staple food for a large part of the world’s human population, especially in Asia and Africa [40,44]. It is an agricultural commodity with the third-highest worldwide production (*O. sativa* was 741.5 million tonnes in 2014), after *Saccharum officinarum* (1.9 billion tonnes) and *Zea mays* (1.0 billion tonnes) [47]. *O. sativa* is a monocot, is normally grown as annual plant, although in the tropical regions it can survive as a perennial and can produce a ratoon for up to 30 years. Moreover, *O. sativa* cultivation is well-suited to countries and regions with low labor costs and high rainfall, as it is labor-intensive to cultivate and requires ample water [42,44,48,49]. *O. sativa* is ranked the second most important cereal crop after wheat worldwide, and China is one of the leading countries for rice production [44,50]. It was reported that Cd can be readily taken up by *O. sativa* and translocated to shoot and then to grains [45,46,51] and *O. sativa* is the staple food for more than half of the world’s population. Thus, Cd can enter into the food chain through *O. sativa* consumption, even at low Cd concentrations in the soils, and cause toxicities to humans [44,51,52,53]. In short, *O. sativa* grown on Cd-contaminated soils challenges food production and quality. Therefore, there is an increasing demand for Cd minimization in rice for the agro-environmental sustainability of world rice production and food safety. However, application of crop residues, manure, compost, fertilizers, micronutrients, and biochars are among organic amendments commonly used under Cd stress [33,38,46]. Selection of low Cd-accumulating *O. sativa* cultivars, crop rotation, planting patterns, water management, application of microbes, and soil types among others, have been successfully employed in reducing Cd uptake by rice [51,53]. Hence, the objective of the present study is to demonstrate the Cd toxicity, tolerance mechanisms, and especially management measures (using Fe fertilizer) for the alleviation of Cd phytotoxicity in *O. sativa*. For this purpose, we designed a hydroponic experiment to study (I) the role of exogenous application of FeSO_4_ on growth and biomass, (II) oxidative stress and antioxidant responses (III) ion uptake and organic acids exudation pattern, and (IV) ultra-structure alterations in the cellular organelles in two Cd-stressed *O*. *sativa* genotypes, i.e., Shan 63 and Lu 9803. The results from the present study gave a new insight that the use of FeSO_4_ in heavy metals studies may be beneficial and can improve plant yield under Cd-contaminated soil.

## 2. Materials and Methods

### 2.1. Experimental Setup

In our previous study, we screened 10 genotypes of rice (*Oryza sativa* L.) under the same levels of Cd stress in a hydroponic environment and found that Shan 63 was sensitive while Lu 9803 was resistant to Cd stress [44]. The seeds of both of these genotypes were surface sterilized with (0.1%) bleaching powder for 10–20 min and washed gently with deionized water and sown in trays. After the emergence of the second leaf, uniform-sized seedlings were transferred to the plastic tubs (4L in volume), which had the Hoagland’s solution (nutrient solution). The chemical composition of Hoagland’s solution used in this study is presented in Table 1. Freshly/newly prepared Hoagland’s solution was changed after every 3 days, and all the plants were monetarized daily. The plastic tubs (label) were arranged at completely randomized design (CRD) with 4 replications with 4 genotypes in a plastic tub (having nutrients solution), and the temperature of the growth chambers (100 W, Guangdong PHILIPS Co., Guangdong, China) was set at 27/30 °C day/night with 70% relative humidity. The pH of all the treatments used in this study was adjusted between 5.5 and 6.5 by using NaOH or HCl during the entire experiment. This hydroponic study was conducted in the growth chambers (under an artificial environment) of Huazhong Agricultural University Wuhan, China (114.20′ E, 30.28′ N). All seedlings were placed under artificial (white) light for 12 h daily. During the entire experiment, we did not observe any symptoms of waterlogging in both genotypes of *O. sativa*. The emerging seedlings remained in the nutrient solution with the treatments of Cd and Fe for 24 days (with 21 days of treatments) and after that time all seedlings were wrapped for various morpho-physio and biochemical traits. All chemicals used were of analytical grade, procured from Sinopharm Chemical Reagent Co., Ltd. (Shanghai, China).

### 2.2. Treatments

After 3 days of shifted *O. sativa* genotypes, all seedlings were treated with various levels of Cd and Fe in the nutrient solution. Cd concentration was provided by using CdCl_2_ at different levels [0 (no Cd), 50 and 100 µM], while Fe concentration was provided by using ferrous sulfate (FeSO_4_) [0 (no Fe), 50 and 100 µM] in the Hoagland’s nutrient solution for 21 days. For each genotype, there was a total of 9 various levels of Cd and Fe and these levels were as follows: (1) 0 µM Cd + 0 µM Fe; (2) 0 µM Cd + 50 µM Fe; (3) 0 µM Cd + 100 µM Fe; (4) 50 µM Cd + 0 µM Fe; (5) 50 µM Cd + 50 µM Fe; (6) 50 µM Cd + 100 µM Fe; (7) 100 µM Cd + 0 µM Fe; (8) 100 µM Cd + 50 µM Fe and (9) 100 µM Cd + 100 µM Fe.

### 2.3. Morphological Traits and Data Collection

The plants were harvested on 15th August 2018 (21 days after the treatment) for the analysis of various morphological parameters. Leaves from each treatment group were picked for chlorophyll, carotenoid, and antioxidant analysis. A fully functional leaf was harvested for the various enzymatic and pigment studies. The leaves were washed with distilled water, placed in liquid nitrogen, and stored at −80 °C for further analysis. The plants from each treatment were washed with tap water to remove debris and waste, and then with distilled water. The morphological measurements, such as total plant length, root length, shoot fresh weight, root fresh weight, shoot dry weight, and root dry weight, were also measured after harvesting the plants. Total plant length was measured from the top of the leaf tips to the bottom of the roots, and root length was also measured in the same way. Plant fresh weight was determined by measuring the weight of the plant with a digital weighting balance. The plant samples were oven-dehydrated at 65 °C for 72 h for Cd and ions concentration determination and the total plant dry weight was also measured. Before being oven-dried, roots were immersed in 20 mM Na_2_EDTA for 15–20 min to remove Cd adhered to the surface of roots. Then, roots were washed thrice with distilled water and finally once with deionized water and dried for further analysis.

### 2.4. Determination of Photosynthetic Pigments and Gas Exchange Parameters

Leaves were collected for the determination of chlorophyll and carotenoid contents. For chlorophylls, 0.1 g of fresh leaf sample was extracted with 8 mL of 95% acetone for 24 h at 4 °C in the dark. The absorbance was measured by a spectrophotometer (UV-2550; Shimadzu, Kyoto, Japan) at 646.6, 663.6, and 450 nm. Chlorophyll content was calculated by the standard method of Arnon [54].

Gas exchange parameters were also measured during the same days. Net photosynthesis (*Pn*), leaf stomatal conductance *(Gs)*, transpiration rate (*Ts*), and intercellular carbon dioxide concentration (*Ci*) were measured from 3 different plants in each treatment group. Measurements were conducted between 11:30 and 13:30 on days with a clear sky. Rates of leaf *Pn*, *Gs, Ts*, and *Ci* were measured with a LI-COR gas-exchange system (LI-6400; LI-COR Biosciences, Lincoln, NE, USA) with a red-blue LED light source on the leaf chamber. In the LI-COR cuvette, CO_2_ concentration was set as 380 mmol mol^−1^ and LED light intensity was set at 1000 mmol m^−2^ s^−1^, which was the average saturation intensity for photosynthesis in *O. sativa* [55].

### 2.5. Determination of Oxidative Stress Indicators

The degree of lipid peroxidation was evaluated as malondialdehyde (MDA) contents. Briefly, 0.1 g of frozen leaves were ground at 4 °C in a mortar with 25 mL of 50 mM phosphate buffer solution (pH 7.8) containing 1% polyethene pyrrole. The homogenate was centrifuged at 10,000× *g* at 4 °C for 15 min. The mixtures were heated at 100 °C for 15–30 min and then quickly cooled in an ice bath. The absorbance of the supernatant was recorded by using a spectrophotometer (xMark™ Microplate Absorbance Spectrophotometer; Bio-Rad, Hercules, CA, USA) at wavelengths of 532, 600, and 450 nm. Lipid peroxidation was expressed as 1 mol g^−1^ by using the formula: 6.45 (A532 − A600) − 0.56 A450. Lipid peroxidation was measured by using a method previously published by Heath and Packer [56].

To estimate H_2_O_2_ content of plant tissues (root and leaf), 3 mL of sample extract was mixed with 1 mL of 0.1% titanium sulfate in 20% (*v*/*v*) H_2_SO_4_ and centrifuged at 6000× *g* for 15 min. The yellow color intensity was evaluated at 410 nm. The H_2_O_2_ level was computed by the extinction coefficient of 0.28 mmol^−1^ cm^−1^. The contents of H_2_O_2_ were measured by the method presented by Jana and Choudhuri [57].

Stress-induced electrolyte leakage (EL) of the uppermost stretched leaves was determined by using the methodology of Dionisio-Sese and Tobita [58]. The leaves were cut into minor slices (5 mm length) and placed in test tubes having 8 mL distilled water. These tubes were incubated and transferred into a water bath for 2 h prior to measuring the initial electrical conductivity (EC_1_). The samples were autoclaved at 121 °C for 20 min and then cooled down to 25 °C before measuring the final electrical conductivity (EC_2_). Electrolyte leakage was calculated by the following formula;

EL = (EC_1_/EC_2_) × 100

### 2.6. Determination of Antioxidant Enzyme Activities

To evaluate enzyme activities, fresh leaves (0.5 g) were homogenized in liquid nitrogen and 5 mL of 50 mmol sodium phosphate buffer (pH 7.0), including 0.5 mmol EDTA and 0.15 mol NaCl. The homogenate was centrifuged at 12,000× *g* for 10 min at 4 °C, and the supernatant was used for measurement of superoxidase dismutase (SOD) and peroxidase (POD) activities. SOD activity was assayed in 3 mL reaction mixture containing 50 mM sodium phosphate buffer (pH 7), 56 mM nitro blue tetrazolium, 1.17 mM riboflavin, 10 mM methionine, and 100 μL enzyme extract. Finally, the sample was measured by using a spectrophotometer (xMark™ Microplate Absorbance Spectrophotometer; Bio-Rad). Enzyme activity was measured by using a method by Chen and Pan [59] and expressed as U g^−1^ FW.

POD activity in the leaves was estimated by using the method of Sakharov and Ardila [60] by using guaiacol as the substrate. A reaction mixture (3 mL) containing 0.05 mL of enzyme extract, 2.75 mL of 50 mM phosphate buffer (pH 7.0), 0.1 mL of 1% H_2_O_2_, and 0.1 mL of 4% guaiacol solution was prepared. Increases in the absorbance at 470 nm because of guaiacol oxidation was recorded for 2 min. One unit of enzyme activity was defined as the amount of the enzyme.

Catalase (CAT) activity was analyzed according to Aebi [61]. The assay mixture (3.0 mL) was comprised of 100 μL enzyme extract, 100 μL H_2_O_2_ (300 mM), and 2.8 mL 50 mM phosphate buffer with 2 mM ETDA (pH 7.0). The CAT activity was measured from the decline in absorbance at 240 nm as a result of H_2_O_2_ loss (*ε* = 39.4 mM^−1^ cm^−1^).

Ascorbate peroxidase (APX) activity was measured according to Nakano and Asada [62]. The mixture containing 100 μL enzyme extract, 100 μL ascorbate (7.5 mM), 100 μL H_2_O_2_ (300 mM), and 2.7 mL 25 mM potassium phosphate buffer with 2 mM EDTA (pH 7.0) was used for measuring APX activity. The oxidation pattern of ascorbate was estimated from the variations in wavelength at 290 nm (*ε* = 2.8 mM^−1^ cm^−1^).

### 2.7. Determination of non-Enzymatic Antioxidants, Sugars, and Proline Contents

Plant ethanol extracts were prepared for the determination of non-enzymatic antioxidants and some key osmolytes. For this purpose, 50 mg of dry plant material was homogenized with 10 mL ethanol (80%) and filtered through Whatman No. 41 filter paper. The residue was re-extracted with ethanol, and the 2 extracts were pooled together to a final volume of 20 mL. The determination of flavonoids [63], phenolics [64], ascorbic acid [65], anthocyanin [66], and total sugars [67] was performed from the extracts.

Fresh leaf material (0.1 g) was mixed thoroughly in 5 mL aqueous sulphosalicylic acid (3%). The mixture was centrifuged at 10,000× *g* for 15 min, and an aliquot (1 mL) was poured into a test tube having 1 mL acidic ninhydrin and 1 mL glacial acetic acid. The reaction mixture was first heated at 100 °C for 10 min and then cooled in an ice bath. The reaction mixture was extracted with 4 mL toluene, and test tubes were vortexed for 20 s and cooled. Thereafter, the light absorbance at 520 nm was measured by using a UV–VIS spectrophotometer (Hitachi U-2910, Tokyo, Japan). The free proline content was determined on the basis of the standard curve at 520 nm absorbance and expressed as µmol (g FW) ^−1^ [68].

### 2.8. Determination of Nutrient Contents

For nutrient analysis, plant roots and shoots were washed twice in redistilled water, dipped in 20 mM EDTA for 3 s, and then, again, washed with deionized water twice for the removal of adsorbed metal on the plant surface. The washed samples were then oven-dried for 24 h at 105 °C. The dried roots and shoots were digested by using a wet digestion method in HNO_3_: HClO_4_ (7:3 V/V) until clear samples were obtained. Each sample was filtered and diluted with redistilled water up to 50 mL. The root and shoot contents of Fe, Mg, and P and were analyzed by using Atomic Absorption Spectrophotometer (AAS) model Agilent 240FS-AA.

### 2.9. Root Exudates Analysis and Cd Contents

In order to determine the concentration of organic acids, freeze-dried exudates were mixed with ethanol (80%), and 20 μL of the solutions were injected into the C18 column (Brownlee Analytical C-183 µm; length 150 mm × 4.6 mm^2^). Quantitative analysis of organic acids in root exudates was executed with high-performance liquid chromatography (HPLC), having a Flexer FX-10 UHPLC isocratic pump (PerkinElmer, Boston, MA, USA). The mobile phase used in HPLC was comprised of an acidic solution of aceto-nitrile containing aceto-nitrile:H_2_SO_4_:acetic acid in ratios of 15:4:1, respectively, and pH of 4.9. The samples were analyzed at a flow rate of 1.0 mL min^−1^ for a time period of 10 min. The inner temperature of the column was fixed at 45 °C, and quantification of organic acids was carried out at 214 nm wavelength with the help of a detector (UV–VIS Series 200, Boston, MA, USA) as described by UdDin et al. [69]. Freeze-dried samples were dissolved in redistilled water, and the pH of the exudates was recorded with LL micro-pH glass electrode by using a pH meter (ISTEK Model 4005–08007 Seoul, South Korea).

Finely ground samples were digested with pure HNO_3_ at 190 °C for 45 min (10 min pre-heating, 15 min heating, 20 min cooling) in a microwave oven (Mars 6, CEM Corporation, Matthews, NC, USA) with the settings described in details by Jezek et al. [70]. Samples were diluted with 2% HNO_3_ and determined by atomic absorption spectrophotometer (AAS) model Agilent 240FS-AA.

### 2.10. Transmission Electron Microscopy

For TEM, leaf samples were collected and placed in liquid nitrogen. Small sections of the leaves (1–3 mm in length) were fixed in 4% glutaraldehyde (*v/v*) in 0.2-mol/L SPB (sodium phosphate buffer, pH 7.2) for 6–8 h and post-fixed in 1% OsO_4_ for 1 h, then in 0.2-mol/L SPB (pH 7.2) for 1–2 h. Samples were dehydrated in a graded ethanol series (50%, 60%, 70%, 80%, 90%, 95%, and 100%) followed by acetone, filtered, and embedded in Spurr resin. Ultra-thin sections (80 nm) were prepared and mounted on copper grids for observation under a transmission electron microscope (JEOL TEM-1200EX) at an accelerating voltage of 60.0 kV or 80.0 kV.

### 2.11. Statistical Analysis

Statistical analysis of data was performed with analysis of variance (ANOVA) by using a statistical program Co-Stat version 6.2, Cohorts Software, 2003, Monterey, CA, USA. All the data obtained were tested by two-way analysis of variance (ANOVA). Thus, the differences between treatments were determined by using ANOVA, and the least significant difference test (*p <* 0.05) was used for multiple comparisons between treatment means. Logarithmic or inverse transformations were performed for data normalization, where necessary, prior to analysis. Pearson’s correlation analysis was performed to quantify relationships between various analyzed variables. The graphical presentation was carried out by using Origin-Pro 2017. The Pearson correlation (heat-map) coefficients between the measured variables of *O. sative* were also calculated using the Rstudio software.

## 3. Results

### 3.1. Effect of Exogenous Application of FeSO_4_ on Plant Growth and Photosynthetic Measurements in O. sativa Genotypes under Toxic Concentrations of Cd

**Figure 1:**
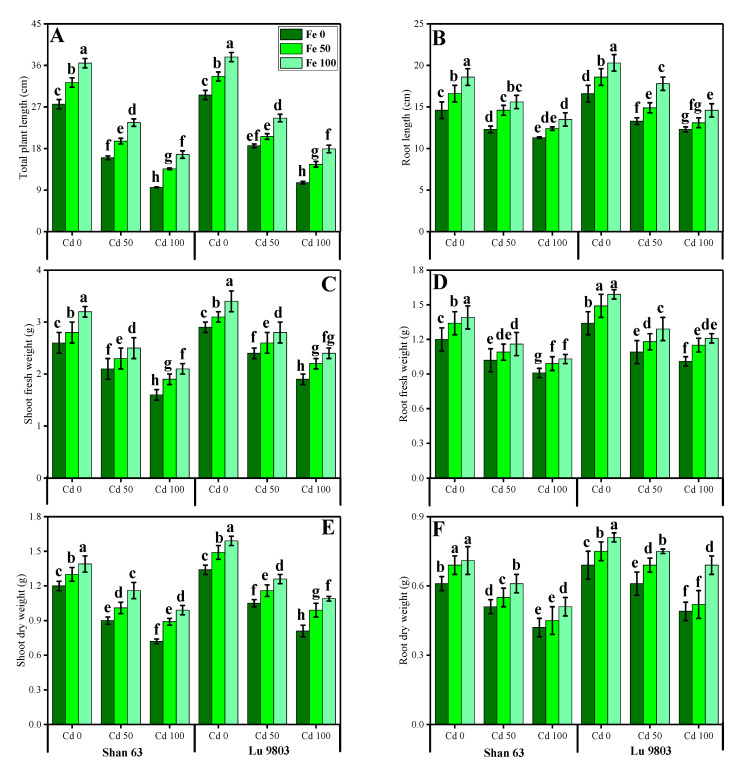
Effect of different concentrations of exogenous application of FeSO_4_ (0, 50, and 100 µM) on morphological attributes, i.e., total plant length (**A**), root length (**B**), shoot fresh weight (**C**), root fresh weight (**D**), shoot dry weight (**E**) and root dry weight (**F**) of *Oryza sativa* (cultivars) grown under different levels of Cd stress (0, 50 and 100 µM). Bars sharing different letter(s) for each parameter are significantly different from each other according to Duncan’s multiple range test (*p* < 0.05). All the data represented are the average of four replications (*n* = 4). Error bars represent the standard deviation (SD) of four replicates.

**Figure 2:**
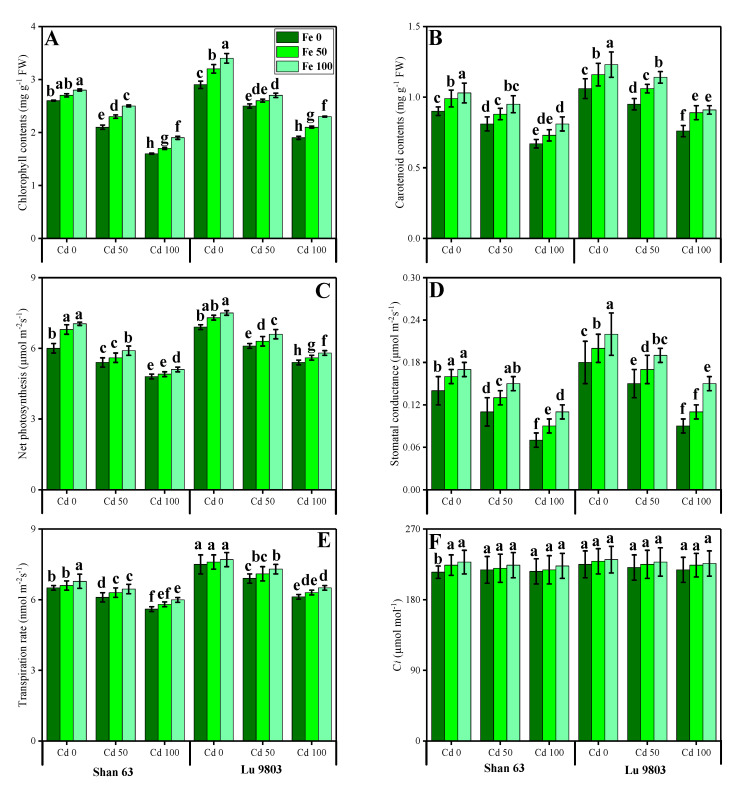
Effect of different concentrations of exogenous application of FeSO_4_ (0, 50, and 100 µM) on photosynthetic pigments and gaseous exchange attributes, i.e., total chlorophyll contents (**A**), carotenoid contents (**B**), net photosynthesis (**C**) stomatal conductance (**D**), transpiration rate (**E**) and intercellular CO_2_ (**F**) of *Oryza sativa* (cultivars) grown under different levels of Cd stress (0, 50 and 100 µM). Bars sharing different letter(s) for each parameter are significantly different from each other according to Duncan’s multiple range test (*p* < 0.05). All the data represented are the average of four replications (*n* = 4). Error bars represent the standard deviation (SD) of four replicates.

In this study, we elucidated various growth parameters, photosynthetic pigments, and gas exchange characteristics under the various levels of FeSO_4_ in Cd-polluted nutrient solution in *O. sativa* genotypes. We presented the various morphological traits of *O. sativa* genotypes in Figure 1 and photosynthetic pigments and gas exchange characteristics in Figure 2, which were grown in Cd-polluted environment under the application of various levels of FeSO_4_. Our results depicted that all total plant length, root length, shoot fresh weight, root fresh weight, shoot dry weight, root dry weight, chlorophyll contents, carotenoid contents, net photosynthesis, stomatal conductance, transpiration rate, and intercellular CO_2_ were decreased with the increase in the Cd levels (50 and 100 µM) in the nutrient solution when compared with the plants grown without the addition of Cd in the nutrient solution in both *O. sativa* genotypes (Figure 1 and Figure 2). In addition, results from the present study also showed that Lu 9803 showed more tolerance/resistance to the Cd stress in the nutrient solution compared to Shan 63 at all levels of Cd-stressed environment in the nutrients solution. We also noticed that various growth parameters, photosynthetic pigments, and gas exchange characteristics could be increased under the toxic concentration of Cd in the nutrient solution by the exogenous application of FeSO_4_ (Figure 1 and Figure 2). In addition, results also showed that exogenous application with FeSO_4_ increased all growth parameters, photosynthetic pigments, and gas exchange characteristics in both genotypes of *O. sativa*, compared to those plants that were grown without the exogenous application with FeSO_4_ in the nutrient solution.

### 3.2. Effect of Exogenous Application of FeSO_4_ on Oxidative Stress Indicators in O. sativa Genotypes under Toxic Concentrations of Cd

**Figure 3:**
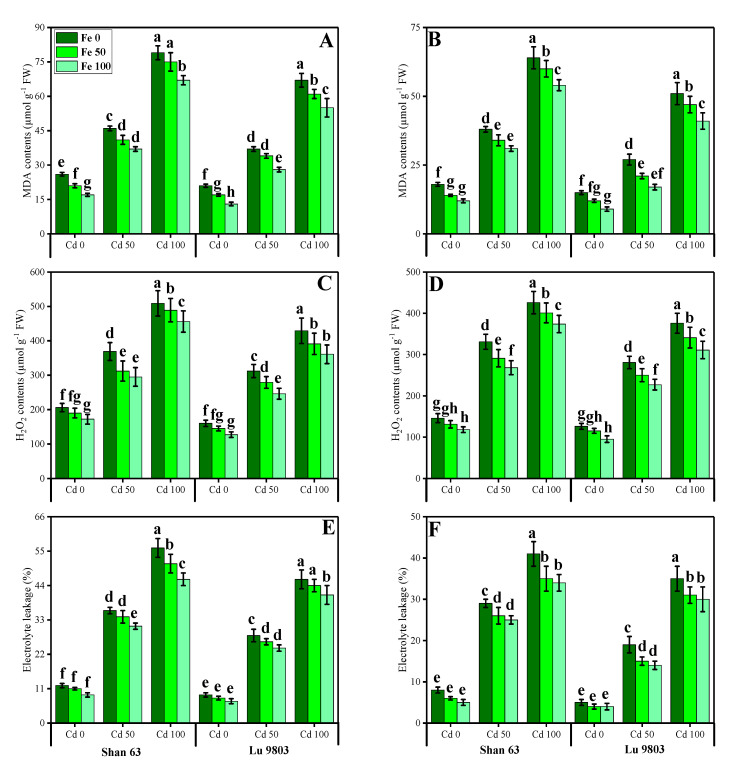
Effect of different concentrations of exogenous application of FeSO_4_ (0, 50, and 100 µM) on oxidative stress indicators, i.e., MDA contents in the roots (**A**), MDA contents in the leaves (**B**), H_2_O_2_ contents in the roots (**C**), H_2_O_2_ contents in the leaves (**D**), EL percentage in the roots (**E**) and EL percentage in the leaves (**F**) of *Oryza sativa* (cultivars) grown under different levels of Cd stress (0, 50 and 100 µM). Bars sharing different letter(s) for each parameter are significantly different from each other according to Duncan’s multiple range test (*p* < 0.05). All the data represented are the average of four replications (*n* = 4). Error bars represent the standard deviation (SD) of four replicates.

Oxidative stress markers, i.e., malondialdehyde (MDA) contents, hydrogen peroxide (H_2_O_2_) initiation, and electrolyte leakage (%) in the roots and leaves of *O. sativa* genotypes grown in toxic concentration of Cd in the nutrient solution, were also measured in the present study. The results regarding MDA, H_2_O_2_, and EL in the roots and leaves of *O. sativa* genotypes grown under the application of FeSO_4_ in Cd-polluted nutrient solution are presented in Figure 3. We also manifested MDA, H_2_O_2_, and EL in the roots and leaves of *O. sativa* genotypes in various toxic concentrations of Cd in the nutrient solution. From the given results, we also elucidated that increasing concentration of Cd in the nutrient solution induced a significant (*p* < 0.05) increase in the contents of MDA, H_2_O_2_ initiation, and EL (%) in the roots and leaves of *O. sativa* genotypes, when compared with those plants, which were grown without the addition of Cd concentration in the nutrient solution (Figure 3). Results also showed that the contents of MDA, H_2_O_2_ initiation and EL (%) were significantly (*p* < 0.05) higher in the roots when compared to the shoots of *O. sativa* genotypes. In addition, the Cd-sensitive genotype, i.e., Shan 63 showed higher values of MDA contents, H_2_O_2_ initiation, and EL (%) in all organs of the plants, in comparison with the Cd-tolerant genotype, i.e., Lu 9803 (Figure 3). Results also illustrated that the application of FeSO_4_ decreased, the contents of MDA, H_2_O_2_ initiation, and EL (%) in the roots and leaves of *O. sativa* genotypes, compared with those plants, which were grown without the exogenous application with FeSO_4_ in the nutrient solution. In addition, at all levels of Cd stress (50 and 100 µM), the contents of MDA, H_2_O_2_ initiation, and EL (%) were decreased with the increasing levels of FeSO_4_ (50 and 100 µM) in the nutrient solution, compared with those plants, which were grown without the application of FeSO_4_.

### 3.3. Effect of Exogenous Application of FeSO_4_ on Enzymatic Antioxidants in O. sativa Genotypes under Toxic Concentrations of Cd

**Figure 4:**
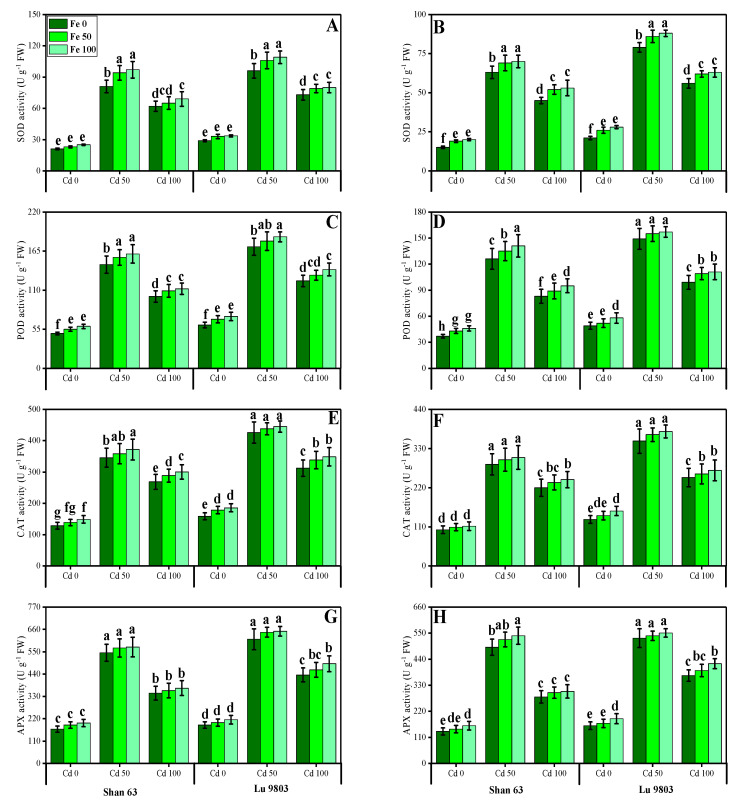
Effect of different concentrations of exogenous application of FeSO_4_ (0, 50, and 100 µM) on antioxidant activities, i.e., SOD activity in the roots (**A**), SOD activity in the leaves (**B**), POD activity in the roots (**C**), POD activity in the leaves (**D**) CAT activity in the roots (**E**), CAT activity in the leaves (**F**), APX activity in the roots (**G**) and APX activity in the leaves (**H**) of *Oryza sativa* (cultivars) grown under different levels of Cd stress (0, 50, and 100 µM). Bars sharing different letter(s) for each parameter are significantly different from each other according to Duncan’s multiple range test (*p* < 0.05). All the data represented are the average of four replications (*n* = 4). Error bars represent the standard deviation (SD) of four replicates.

In the present study, we also measured various enzymatic antioxidants, i.e., superoxidase dismutase (SOD), peroxidase (POD), catalase (CAT), and ascorbate peroxidase (APX) from the roots and leaves of *O. sativa* genotypes grown under the application of FeSO_4_ in Cd-polluted nutrient solution. The data regarding the activities of antioxidants (SOD, POD, CAT, and APX) in the roots and leaves of *O. sativa* genotypes grown under the application of FeSO_4_ in Cd-polluted nutrient solution are presented in Figure 4. According to the given results, we elucidated that increasing concentrations of Cd in the nutrient solution increased activities of antioxidants (SOD, POD, CAT, and APX) in the roots and leaves of *O. sativa* genotypes, compared with those plants, which were grown without the addition of Cd in the nutrient solution. The activities of various antioxidants (SOD, POD, CAT, and APX) in the roots and leaves of *O. sativa* genotypes initially increased up to a Cd level of 50 µM in the nutrient solution, but further increase in Cd concentration in the nutrient solution (100 µM) induced a significant (*p* < 0.05) decrease in antioxidants in the roots of leaves of both *O. sativa* genotypes (Figure 4). Results also showed that the activities of antioxidants (SOD, POD, CAT, and APX) were significantly (*p* < 0.05) higher in the roots when compared to the shoots of *O. sativa* genotypes. In addition, the Cd-sensitive genotype, i.e., Shan 63 showed higher values of the activities of antioxidants (SOD, POD, CAT, and APX) in all organs of the plants, in comparison with Cd-tolerant genotype, i.e., Lu 9803 (Figure 4). Results also illustrated that the application of FeSO_4_ increased non-significantly (*p* < 0.05) the activities of antioxidants (SOD, POD, CAT, and APX) in the roots and leaves of *O. sativa* genotypes, compared with those plants, which were grown without the exogenous application with FeSO_4_ in the nutrient solution (Figure 4). In addition, at all levels of Cd stress (50 and 100 µM), the activities of antioxidants (SOD, POD, CAT, and APX) were increased with the increasing levels of FeSO_4_ (50 and 100 µM) in the nutrient solution, compared with those plants, which were grown without the application of FeSO_4_ (Figure 4).

### 3.4. Effect of Exogenous Application of FeSO_4_ on Non-Enzymatic Antioxidants, Sugars and Proline in O. sativa Genotypes under Toxic Concentrations of Cd

**Figure 5:**
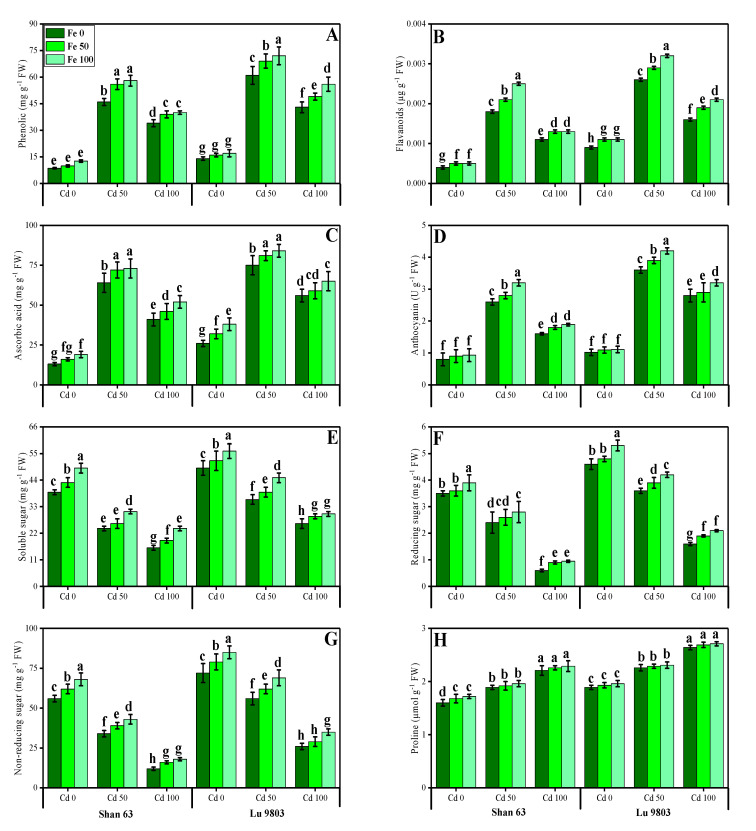
Effect of different concentrations of exogenous application of FeSO_4_ (0, 50, and 100 µM) on non-enzymatic antioxidants and sugars, i.e., phenolic contents (**A**), flavonoid contents (**B**), ascorbic acid contents (**C**), anthocyanin contents (**D**), soluble sugar contents (**E**), reducing sugar contents (**F**), non-reducing sugar contents (**G**) and proline contents (**H**) in the leaves of *Oryza sativa* (cultivars) grown under different levels of Cd stress (0, 50, and 100 µM). Bars sharing different letter(s) for each parameter are significantly different from each other according to Duncan’s multiple range test (*p* < 0.05). All the data represented are the average of four replications (*n* = 4). Error bars represent the standard deviation (SD) of four replicates.

In the present study, we also determined the contents of non-enzymatic antioxidant compounds (phenolics, flavonoids, ascorbic acid, and anthocyanin) from the leaves of *O. sativa* genotypes under the application of FeSO_4_, grown in Cd-polluted nutrient solution. The data regarding the non-enzymatic antioxidant compounds (phenolics, flavonoids, ascorbic acid, and anthocyanin) from the leaves of *O. sativa* genotypes grown under the application of FeSO_4_ in Cd-polluted nutrient solution are presented in Figure 5. These results showed that the increasing concentration of Cd in the nutrient solution induced a significant increase in the contents of non-enzymatic antioxidants in the leaves of O. sativa genotypes, with the application of FeSO_4_, grown in Cd-polluted nutrient solution (Figure 5). The compounds of non-enzymatic antioxidant compounds (phenolics, flavonoids, ascorbic acid, and anthocyanin) in the leaves of *O. sativa* genotypes initially increased up to a Cd level of 50 µM in the nutrient solution, but further increase in Cd concentration in the nutrient solution (100 µM) induced a significant (*p* < 0.05) decrease in antioxidants in the roots or leaves of both *O. sativa* genotypes (Figure 5). In addition, the Cd-sensitive genotype, i.e., Shan 63 showed higher contents non-enzymatic antioxidant compounds in the leaves of the plants, in comparison with the Cd-tolerant genotype, i.e., Lu 9803 (Figure 4). Results also illustrated that the application of FeSO_4_ increased non-significantly (*p* < 0.05) the non-enzymatic antioxidant compounds (phenolics, flavonoids, ascorbic acid, and anthocyanin) in the leaves of *O. sativa* genotypes, compared with those plants, which were grown without the exogenous application with FeSO_4_ in the nutrient solution (Figure 5). In addition, at all levels of Cd stress (50 and 100 µM), the non-enzymatic antioxidant compounds were increased with the increasing levels of FeSO_4_ (50 and 100 µM) in the nutrient solution, compared with those plants, which were grown without the application of FeSO_4_ (Figure 4).

The contents of sugars (soluble, reducing, and non-reducing) and proline in the leaves of *O. sativa* genotypes grown in the external application with FeSO_4_ in the Cd-polluted nutrient solution are also presented in Figure 5. It was also observed that the contents of soluble, reducing, and non-reducing sugars were decreased with the increasing concentration of Cd (50 and 100 µM) in the nutrient solution without the application of FeSO_4_. The contents of soluble, reducing, and non-reducing sugars were decreased significantly (*p* < 0.05) under all levels of Cd toxicity (50 and 100 µM) in the nutrient solution while increasing Cd concentration induced a significant (*p* < 0.05) increase in proline contents in the leaves of *O. sativa* genotypes, compared to those plants, which were grown without the addition of Cd in the nutrient solution. In addition, the Cd-sensitive genotype, i.e., Shan 63 showed lower contents of soluble, reducing, and non-reducing sugars in the leaves of the plants, in comparison with the Cd-tolerant genotype, i.e., Lu 9803 (Figure 5). Results also illustrated that the application of FeSO_4_ increased non-significantly (*p* < 0.05) the contents of soluble, reducing, and non-reducing sugars and the contents of proline in the leaves of *O. sativa* genotypes, compared with those plants, which were grown without the exogenous application with FeSO_4_ in the nutrient solution (Figure 5). In addition, at all levels of Cd stress (50 and 100 µM), the soluble, reducing, and non-reducing sugars and the contents of proline were increased with the increasing levels of FeSO_4_ (50 and 100 µM) in the nutrient solution, compared with those plants, which were grown without the application of FeSO_4_ (Figure 5).

### 3.5. Effect of Exogenous Application of FeSO_4_ on Nutrients Uptake in O. sativa Genotypes under Toxic Concentrations of Cd

**Figure 6:**
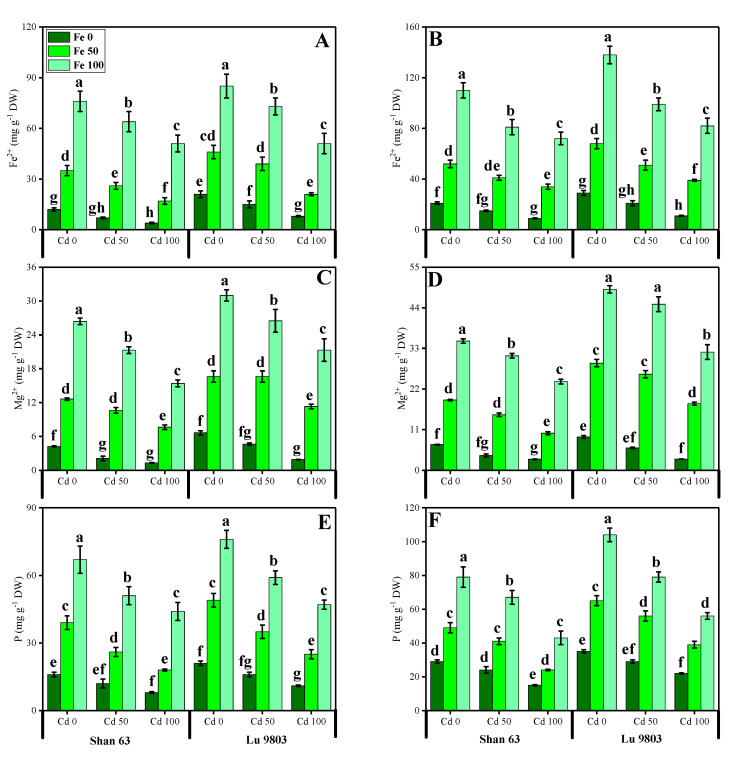
Effect of different concentrations of exogenous application of FeSO_4_ (0, 50, and 100 µM) on ion uptake, i.e., iron contents in the roots (**E**), iron contents in the shoots (**F**), calcium contents in the roots (**G**), and calcium contents in the leaves (**H**), magnesium contents in the roots (**A**), magnesium contents in the shoots (**B**), phosphorus contents in the roots (**C**) and phosphorus contents in the shoots (**D**) of *Oryza sativa* (cultivars) grown under different levels of Cd stress (0, 50 and 100 µM). Bars sharing different letter(s) for each parameter are significantly different from each other according to Duncan’s multiple range test (*p* < 0.05). All the data represented are the average of four replications (*n* = 4). Error bars represent the standard deviation (SD) of four replicates.

In the present study, the contents of essential minerals, i.e., iron (Fe^2+^), magnesium (Mg^2+^), and phosphorus (P) were also determined from the roots and shoots of *O. sativa* genotypes grown in different application levels of FeSO_4_ (50 and 100 µM) under Cd-polluted nutrient solution. The contents of Fe^2+^, Mg^2+^, and P from the roots and shoots of *O. sativa* genotypes are presented in Figure 6. Our results depicted that the concentrations of Fe^2+^, Mg^2+^, and P in the roots and shoots of *O. sativa* genotypes were decreased with the increase in the Cd levels (50 and 100 µM) in the nutrient solution, when compared with the plants grown without the addition of Cd in the nutrient solution in both *O. sativa* genotypes (Figure 6). In addition, results from the present study also showed that Lu 9803 showed more concentrations of Fe^2+^, Mg^2+^, and P in the roots and shoots of the plants compared to Shan 63 at all levels of Cd-stressed environment in the nutrients solution. We also noticed that the concentrations of Fe^2+^, Mg^2+^, and P in the roots and shoots of *O. sativa* genotypes could be increased under the toxic concentration of Cd in the nutrient solution by the exogenous application of FeSO_4_ (Figure 6). In addition, results also showing that exogenous application with FeSO_4_ increased the concentrations of Fe^2+^, Mg^2+^, and P in the roots and shoots of *O. sativa* genotypes, compared to those plants, which were grown without the exogenous application with FeSO_4_ in the nutrient solution.

### 3.6. Effect of Exogenous Application of FeSO_4_ on Organic Acids Exudation and Cd Uptake and Accumulation in O. sativa Genotypes under Toxic Concentrations of Cd

**Figure 7:**
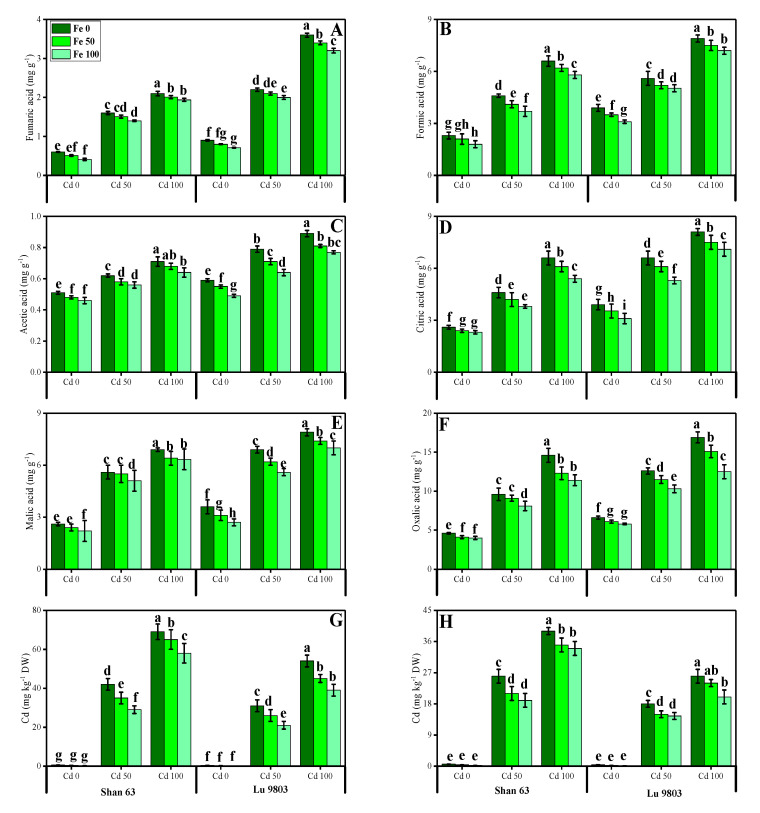
Effect of different concentrations of exogenous application of FeSO_4_ (0, 50, and 100 µM) on fumaric acid contents (**A**), formic acid contents (**B**), acetic acid contents (**C**), citric acid contents (**D**), malic acid contents (**E**), oxalic acid contents (**F**) in the roots and Cd concentration in the roots (**G**) and Cd concentration in the shoots (**H**) of *Oryza sativa* (cultivars) grown under different levels of Cd stress (0, 50 and 100 µM). Bars sharing different letter(s) for each parameter are significantly different from each other according to Duncan’s multiple range test (*p* < 0.05). All the data represented are the average of four replications (*n* = 4). Error bars represent the standard deviation (SD) of four replicates.

The contents of fumaric acid, formic acid, acetic acid, citric acid, malic acid, and oxalic acid in the roots of both genotypes of *O. sativa* grown under toxic levels of Cd in the nutrient solution, with or without the application of FeSO_4_ are presented in Figure 7. According to the given results, we have noticed that increasing the concentration of Cd in the nutrient solution (50 and 100 µM) induced a significant (*p* < 0.05) increase in the contents of fumaric acid, formic acid, acetic acid, citric acid, malic acid, and oxalic acid in the roots of both genotypes of *O. sativa*, compared to those plants, which were grown in Cd level of 0 µM in the nutrient solution. In addition, the Cd-sensitive genotype, i.e., Shan 63 showed higher contents of fumaric acid, formic acid, acetic acid, citric acid, malic acid, and oxalic acid, in comparison with the Cd-tolerant genotype, i.e., Lu 9803 (Figure 7). Results also illustrated that the application of FeSO_4_ decreased the contents of fumaric acid, formic acid, acetic acid, citric acid, malic acid, and oxalic acid in the roots of *O. sativa* genotypes, compared with those plants, which were grown without the exogenous application with FeSO_4_ in the nutrient solution. In addition, at all levels of Cd stress (50 and 100 µM), the contents of fumaric acid, formic acid, acetic acid, citric acid, malic acid, and oxalic acid decreased with the increasing levels of FeSO_4_ (50 and 100 µM) in the nutrient solution, compared with those plants, which were grown without the application of FeSO_4_.

We also manifested the contents of Cd from the roots and shoots of *O. sativa* genotypes grown under toxic levels of Cd in the nutrient solution, with or without the application of FeSO_4_ are presented in Figure 7. Increasing levels of Cd in the nutrient solution induced a significant (*p* < 0.05) increase in the Cd concentration in the roots and shoots of *O. sativa* genotypes, compared to those plants which were grown in the control treatment. In addition, the Cd-sensitive genotype, i.e., Shan 63 showed higher contents of Cd in the roots and shoots of the plants, in comparison with the Cd-tolerant genotype, i.e., Lu 9803 (Figure 7). In addition, at all levels of Cd stress (50 and 100 µM), the contents of Cd were decreased with the increasing levels of FeSO_4_ (50 and 100 µM) in the nutrient solution, compared with those plants, which were grown without the application of FeSO_4_.

### 3.7. Effect of Exogenous Application of FeSO_4_ on Transmission Electron Microscopy in O. sativa Genotypes under Toxic Concentrations of Cd

**Figure 8:**
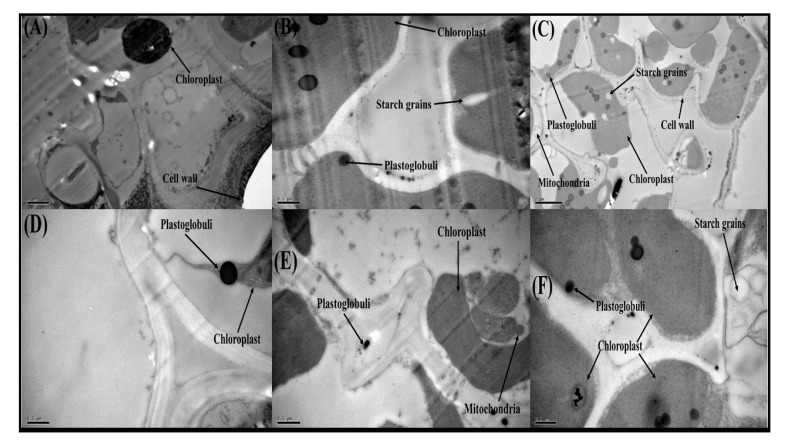
Transmission electron microscopy (TEM) analysis of *Oryza sativa* (cultivars) leaf structure after treated with Cd and Fe concentration in the nutrient solution. (**A**) Shan 63 (5000) with Cd 100 + Fe 0, (**B**) Shan 63 (2500) with Cd 100 + Fe 50, (**C**) Shan 63 (10,000) with Cd 100 + Fe 100, (**D**) Lu 9803 (2500) Cd 100 + Fe 0, (**E**) Lu 9803 (2500) Cd 100 + Fe 50 and (**F**) Lu 9803 (2500) Cd 100 + Fe 100.

In the section of leaf samples of *O. sativa* genotypes with the Cd concentration in the nutrient solution (100 µM), the Cd toxicity destroyed most of the cellular organelles such as chloroplasts, plastoglobuli, mitochondria, starch grains, and the cell wall in both genotypes of *O. sativa*. Furthermore, Figure 8 is also showing that there was serious damage in all membrane-bounded organelles in both genotypes of *O. sativa*, but the Cd-sensitive genotype, i.e., Shan 63 showed more damage to the membrane-bounded structures compared to the Cd-tolerant genotype, i.e., Lu 9803 and also displaying that most of membrane-bounded organelles were dislocated or not visible in TEM study. Although, application with FeSO_4_ recover the cell damage by improving the structure of many membrane-bounded organelles, which are mostly visible under the exogenous supplementation with FeSO_4_.

### 3.8. Correlation between Different Growth, Photosynthetic, Ions Uptake with the Cd Uptake and Accumulation in Various Parts of the Plants

**Figure 9:**
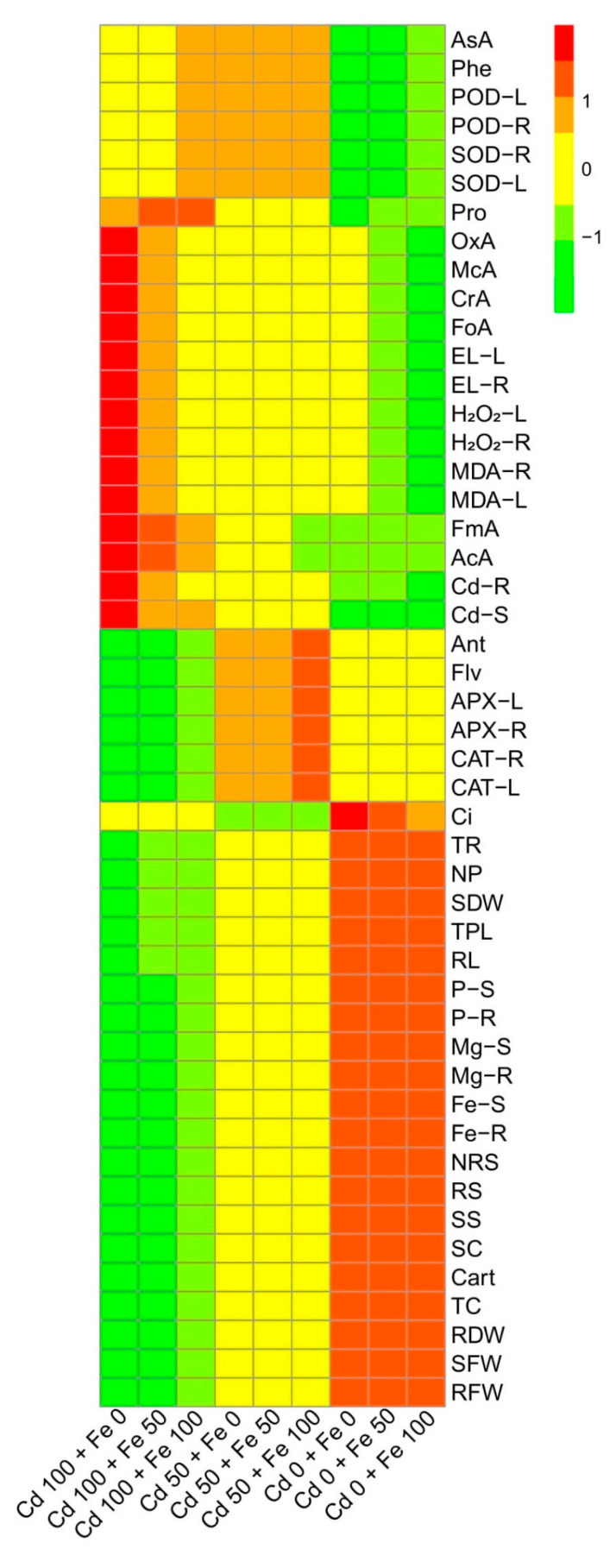
Heatmap histogram correlation between Cd uptake/accumulation with different morpho-physio-biochemical attributes of *Oryza sativa* grown under different levels of Cd stress (0, 50, and 100 µM) with different concentrations of exogenous application of FeSO_4_ (0, 50, and 100 µM). Different abbreviations used are as follow: Ci (intercellular CO_2_), P-S (phosphorus contents in the shoots), P-R (phosphorus contents in the roots), Mg-S (magnesium contents in the shoots), Mg-R (magnesium contents in the roots), Ca-S (calcium contents in the shoots), Ca-R (calcium contents in the roots), Fe-S (iron contents in the shoots), Fe-R (iron contents in the roots), NRS (non-reducing sugars), RS (reducing sugars), SS (soluble sugars), TR (transpiration rate), SC (stomatal conductance), NP (net photosynthesis), Carot (carotenoid contents), TC (total chlorophyll), RDW (root dry weight), SDW (shoot dry weight), RFW (root fresh weight), SFW (shoot fresh weight), TPL (total plant length), RL (root length), Ant (anthocyanin contents), AsA (ascorbic acid contents), Flv (flavonoid contents), Phe (phenolic contents), APX-L (ascorbate peroxidase activity in the leaves), APX-R (ascorbate peroxidase activity in the roots), CAT-L (catalase activity in the leaves), CAT-R (catalase activity in the roots), POD-R (peroxidase activity in the roots), POD-L (peroxidase activity in the leaves), SOD-R (superoxidase dismutase activity in the roots), SOD-L (superoxidase dismutase activity in the leaves), Pro (proline contents), OxA (oxalic acid contents), McA (melic acid contents), CrA (citric acid contents), AcA (acetic acid contents), FoA (formic acid contents), FmA (fumaric acid contents), EL-L (electrolyte leakage in the leaves), EL-R (electrolyte leakage in the roots), H_2_O_2_-L (hydrogen peroxide initiation in the leaves), H_2_O_2_-R (hydrogen peroxide initiation in the roots), MDA-R (malondialdehyde contents in the roots), MDA-L (malondialdehyde contents in the leaves), Cd-R (Cd concentration in the roots), and Cd-S (Cd concentration in the shoots).

We also constructed histogram-correlation analysis to depict a relationship between *O. sativa* growth, photosynthetic pigments, gas exchange attributes, antioxidant response, nutrients uptake, and organic acids exudation with Cd uptake in the roots and shoots of the plants (Figure 9). Although both genotypes showed the same trend, we constructed only one graph (histogram-correlation analysis) of Shan 63. The significant differences were observed in the plant growth, photosynthetic apparatus, nutrient uptake, and sugar contents in the treatments, which were not spiked artificially with Cd (comprised with the application of FeSO_4_). While the rest of the heat-map showed non-significant results with all other parameters with Cd treatments in the nutrient solution. However, the red color showed non-significant differences within the treatments, while the purple color depicted a significant difference in the histogram study. This histogram study showed clear differences in Cd toxicity on the ecophysiology of *O. sativa* (Shan 63) under the application of FeSO_4_ in the nutrient solution.

## 4. Discussion

Residues from smelting industries, metalliferous mines, oil burning in automobiles, tire dust, Cd containing batteries, polyvinyl plastic, canned foods, metal ice trays, meat processing, wrappings bags, and municipal sewage incineration are sources of Cd pollution that ends up in the wastewater [2,9,19,25,52]. The fact that Cd is non-vital for plant development, but is easily taken up by plant roots and shoots, makes it a metal of utmost concern regarding its accumulation in the food chain [2,22,24,26,28,29]. Photosynthesis, respiration, cell division, water relations, opening and closing of stomata, nitrogen metabolism, and mineral nutrition are the main metabolic processes within the plants, which are negatively affected by Cd stress [14,21,26,52]. Cd reduces the photosynthetic capacity of plants by devastating the enzymes of the Calvin cycle and carbohydrate metabolism and also modulates the antioxidant machinery of the plants. All these physiological changes result in decreased plant yield [8,10,53,71]. Cadmium is indirectly involved in the biological redox reaction, and the oxidative burst is produced by increasing the activity of NADPH oxidases, which results in the production of extracellular superoxide, peroxide, and intracellular lipid peroxidation [44,71]. The gas exchange constitutes an important parameter for evaluation of the photosynthetic activity, which is crucial to determine the adaptation and stability of plants subjected to different environmental adversities, as the variation in photosynthetic rates implies alterations in growth as well as productivity [12,72,73]. Furthermore, Gas exchange characteristics are considered as effective physiological indicators, which could be used to assess the intensity of stress on plants grown under metal toxicity [74,75]. Excessive Cd concentrations affected the net photosynthesis due to two important factors; (i) stomatal factors and (ii) non-stomatal factors. Ascorbic acid-mediated, the closure of the stomata under the excess concentration of Cd causes a reduction in stomatal numbers under the influence of stomatal factors [76,77]. Non-stomatal considerations, however, include limitations of net photosynthesis, probably due to the reduction of different enzymes involved in chlorophyll synthesis, as well as Calvin cycle inhibition, and also phosphoenolpyruvate carboxylase in C4 plants [78,79]. The decrease in growth-related attributes in *O. sativa* was also reported in many studies [46,50,51,53]. This might be linked with various toxicity mechanism in *O. sativa* [1,29,44], which we also found in the present study (Figure 1). We also manifested that, under the same stress levels of Cd (50 and 100 µM), Lu 9803 was more tolerant to Cd stress than Shan 63, which we also found in our previous study [44] under elevating levels of Cd in the nutrient solution. The differences in growth in different genotypes of *O. sativa* under the same stress condition might be due to the low availability of water contents, poor stomatal conductance, and alterations in root architecture [44,80]. It was reported that the low concentration of Cd in the environment could disturb many metabolic pathways such as the rate of photosynthetic and chlorophyll pigments by decreasing their contents/rate in the plants [2,16,20,23], which ultimately decrease crop yield (Figure 1).

Heavy metals are considered a primary source of injury to the cell membrane, frequently attributing to lipid peroxidation. As a result of metal accumulation, a large number of active free oxygen radicals are formed, which may be the main cause of cell membrane lipid peroxidation, and also harm the functioning and structure of the cell membrane [3,4,6,20,81]. Excessive reactive oxygen species (ROS) production causes oxidative stress, as reported for many crops under heavy metals treatment, and is likely to be commenced by molecular oxygen excitation (O_2_) to generate singlet oxygen or by electron transfer to O_2_ and genesis of free radicals, i.e., O^2−^ and OH^−^ [80,82]. Plant response to oxidative stress also depends upon plant species and cultivars, and this ROS production in plants is removed by a variety of antioxidant enzymes such as SOD, POD, CAT, and APX [2,15,44,71]. The increase in the activities of antioxidant enzymes was concomitant with the generation of extra ROS. It was also reported that an increase in the activities of various antioxidant enzymes under environmental stress conditions is also due to the reduction in glutathione contents [2,9,10,21,22]. However, the reduction in antioxidants under severe levels of Cd in soil might be due to alterations in gene expression and function of various proteins in plant tissues [2,26,83]. Plants produce a variety of secondary metabolites such as proline, flavonoids, and phenolics that improve tolerance against metal toxicity [7,23,30,34,52]. Phenolics are potent antioxidants against metal-induced oxidative damage, efficiently scavenge ROS, and are also involved in metal chelation [84]. Flavonoids act as an antioxidant by donating hydrogen atoms and thereby significantly enhancing plant metal tolerance. Higher flavonoids are positively associated with plant stress tolerance [9]. Ascorbic acid is an important non-enzymatic antioxidant compound that significantly mediates metal toxicity in plants [84]. Although proline accumulation in plant tissue/organs is a response to metal toxicity, which might be associated with signal transduction and prevents membrane distortion [2,36,44]. Previously, an increase in antioxidant activities under elevated levels of Cd in the soil/medium was found in *Trachyspermum ammi* [2], *O. sativa* [46], *Pfaffia glomerata* [28], *Solanum lycopersicum* [27] and *Brassica napus* [85]. The decrease in antioxidants at extremely high Cd concentration in the nutrient solution (100 µM) might be due to alterations in gene expression and functions of some proteins in different plant organs [14,25,52]. Similar findings we also observed in our previous study that increasing levels of Cd (1.5 mM) in the sand increased the activities of enzymatic and non-enzymatic antioxidants, but a further increase in the Cd concentration in the sand (3 mM) decreased the compounds of various enzymatic and non-enzymatic antioxidant compounds [2]. The increase in the activities of antioxidant enzymes was concomitant with the generation of extra ROS (Figure 4 and Figure 5). It was also reported that an increase in the activities of various antioxidant enzymes under environmental stress conditions was also due to the reduction in glutathione contents [30,86]. Similar results were observed by Saleem et al. [11] while studying *Hibiscus cannabinus* under various levels of copper and reported that the activities of SOD, POD, CAT and APX were initially increased up to Cu level of 120 μmol L^−1^ while higher Cu level (180 μmol L^−1^) caused a significant (*p <* 0.05) decrease in the activities of antioxidants in roots and shoots.

Essential nutrients are required for the normal growth of plants. Numerous reports demonstrated that the uptake and translocation of essential elements in plants were restricted under Cd stress [39,52,87]. Excess Cd decreased the Fe, Mg, and P contents in numerous plant species, which may cause ions deficiency in plants. It is well known that Cd toxicity in crops depends on the bioavailability of Cd in soils and the concentration of elements, which can compete with Cd during plant uptake [88]. In general, *O. sativa* takes up Cd in the form of Cd^2+^ from the soils. Cd uptake in *O. sativa* plants varies with soil pH and organic matter content present in the soils [89]. After absorption by the roots, Cd is transported to the stele by passing through endodermis, and Casparian strips, and then Cd is translocated to shoot via xylem and finally accumulates in grains [52]. In the present study, increasing concentrations of Cd in the nutrient solution induces a significant (*p <* 0.05) increase in Cd concentration in the roots and shoots of *O. sativa* genotypes (Figure 7) while inducing a significant decrease in the uptake of essential nutrients from the roots and shoots of the *O. sativa* genotypes (Figure 6). These findings coincide with the results obtained by Liu et al. [53] in *O. sativa*, that Cd toxicity increased Cd contents in various parts of plants, which induced deficiency of various essential nutrients in the plants, which are necessary for normal growth and development. Similar findings were also shown by Lin et al. [90] in *O. sativa*. As reported by Rizwan et al. [52], Cd toxicity in the soil decreased plant growth by increasing ROS production and Cd contents in the plant parts, which makes the plant unable to uptake essential ions from the soil. The decrease in essential nutrients in the soil is the main factor that plants decrease their growth (Figure 1) and photosynthesis (Figure 2) and increasing oxidative damage to the membrane-bounded organelles, which was reported in the present study. Roots exclude especially organic acids, which are regarded as active ligands under the excess concentration of metals in the soil [91]. Acidification of mucilage after uptake of Cd is likely due to the release of protons when plant roots release more cations than anions in order to maintain their charge balance [92]. The exudation of organic acids in the roots of *O. sativa* genotypes (Figure 7), accelerating metal transport from roots to the aboveground parts, is possibly due to the formation of metal-chelated ions as suggest by Javed et al. [71], when they cultivated *Zea mays* in Cd-polluted soil. Thus, increasing the contents of organic acids in the roots of *O. sativa* genotypes could be a detoxification mechanism and helps in ameliorates Cd-toxicity in the plants (Figure 7). The cell wall is considered as the primary site for heavy metal accumulation/deposition under severe heavy metal stress conditions. Previously, [93,94] noticed that Cd was extremely amassed in the cell wall of *Scopelophila cataractae* and *Oryza sativa*. However, using TEM-technology, it was revealed that excess Cd mainly affected many membrane-bounded organelles of *O. sativa* genotypes, (Figure 8). It was reported that toxic concentration of Cd in the soil disturbed the ultra-structure of membranous bounded organelles and caused structural damage to photosynthetic apparatus, which ultimately decreased the photosynthetic apparatus in *O. sativa*, when it grows under toxic level of Cd in the soil as discussed in detail in the review of the literature by Rizwan et al. [52].

Different practices have been used for the management of Cd-contaminated soils and its reduction in crop plants [2,13]. There are several studies that reported that the application of elements such as iron (Fe), nitrogen (N), zinc (Zn), selenium (Se), and phosphorus (P) could decrease Cd uptake and toxicity in *O. sativa* [46,52]. Fe is an essential micronutrient for all living organisms, including plants. It plays a significant role in the plant’s physiological and biological function [43]. Fe has been found beneficial regarding its role in reducing heavy metals toxicity in various plants. It was observed that Fe declined Cd toxicity by enhancing plant growth, photosynthetic pigments, and chloroplast quality in almond seedlings, as documented by Wen et al. [41]. Furthermore, Fe application reduced oxidative stress induced by Cd stress and maintains stability in the chloroplast, chlorophyll contents, and thylakoid complexes in Indian mustard [95]. Photosynthetic efficiency and its functioning are largely Fe dependent and under metal stress conditions Fe is involved in the electron transfer chain in thylakoid membranes where they operate as a cofactor of protein complexes [96]. This is because Fe is an essential micronutrient for proper plant growth and development, and it plays a crucial role in plant metabolism in enzymatic and metabolic processes [33,38,43]. Many approaches have been used to alleviate heavy metal such as Cd toxicity in the soil, but the use of Fe provides a direct approach, which not only increases plant yield but also decreases the Cd contents in the plant organs [39,42,46,97]. It is well-documented that Fe can increase crop yield and other physiological attributes by minimizing the metals uptake by the plants when grown in metals contaminated soil [42,97,98]. Previously, Nada et al. [98] reported that the application of Fe increases plant growth and biomass, gas exchange characteristics, and decreases oxidative stress and Cd concentration in the various organs of *Prunus dulcis*, when grown in Cd-contaminated soil. Moreover, similar findings were also observed by Qureshi et al. [95], when they studied *Brassica juncea* under various levels of Cd toxicity and noticed that Fe application maintains chloroplast structure and other membrane-bounded organelles and maintain photosynthetic pigments under Cd toxicity. However, variable antioxidative enzymes in *O. sativa* genotypes might be due to alterations in gene expression and function of some important proteins in different parts of the plants [38]. Although, the increase in antioxidative activities under the application of Fe suggested that plant has better defense system to overcome the production of ROS, generated by Cd toxicity in the nutrient solution [33,99]. This is because of Fe application increases plant growth and biomass by fortifying essential nutrients and decrease Cd concentration in the plant organs as suggested by Bashir et al. [42].

## 5. Conclusions

On the basis of these findings, it can be concluded that the negative impact of Cd toxicity can be overcome by the external application with FeSO_4_. Our results depict that Cd toxicity induced severe metal toxicity in *O. sativa* genotypes by increasing the generation of ROS in the form of oxidative stress and also increased the concentration of Cd in the roots and shoots of the plants. Furthermore, Cd toxicity also increased organic acids exudation and imbalance the nutritional status of the plants, which ultimately decrease plant growth and yield and photosynthetic efficiency. Hence, Cd toxicity was eliminated by the external application of FeSO_4_, which also decreased the Cd concentration in the plant tissues, degenerated ROS, induced ultra-structure alterations and organic acids exudation, and increased the activities of antioxidants and essential nutrients in the plants. Therefore, long-term field studies should be executed to draw parallels amongst plants/crops root exudations, metal stress, Fe fertigation regimes, nutrients mobility patterns, and plant growth in order to gain insights into underlying mechanisms.

## Figures and Tables

**Table 1:** Chemical composition and proportions of Hoagland’s solution used in the hydroponic.

Proportions	Salts
269.76	Ca (NO_3_)_2_·4H_2_O
35.15	KH_2_PO_4_
48.80	K_2_SO_4_
167.68	CaCl_2_·2H_2_O
324.5	MgSO_4_·7H_2_O
2.8818	MnCl_2_·4H_2_O
0.1472	(NH_4_)6Mo_7_O_24_·4H_2_O
1.8304	H_3_BO_3_
0.0704	ZnSO_4_·7H_2_O
13.33	Na_2_EDTA
9.96	FeSO_4_·7H_2_O
0.0629	CuSO_4_·5H_2_O
